# Hemoglobin E Prevalence among Ethnic Groups Residing in Malaria-Endemic Areas of Northern Thailand and Its Lack of Association with *Plasmodium falciparum* Invasion *In Vitro*

**DOI:** 10.1371/journal.pone.0148079

**Published:** 2016-01-25

**Authors:** Pathrapol Lithanatudom, Jiraprapa Wipasa, Pitsinee Inti, Kriangkrai Chawansuntati, Saovaros Svasti, Suthat Fucharoen, Daoroong Kangwanpong, Jatupol Kampuansai

**Affiliations:** 1 Department of Biology, Faculty of Science, Chiang Mai University, Chiang Mai, Thailand; 2 Research Institutes for Health Sciences, Chiang Mai University, Chiang Mai, Thailand; 3 Thalassemia Research Center, Institute of Molecular Biosciences, Mahidol University, Nakornpathom, Thailand; National University of Singapore, SINGAPORE

## Abstract

Hemoglobin E (HbE) is one of the most common hemoglobin variants caused by a mutation in the β-globin gene, and found at high frequencies in various Southeast Asian groups. We surveyed HbE prevalence among 8 ethnic groups residing in 5 villages selected for their high period malaria endemicity, and 5 for low endemicity in northern Thailand, in order to uncover factors which may affect genetic persistence of HbE in these groups. We found the overall HbE prevalence 6.7%, with differing frequencies from 0% in the Pwo Karen, the Lawa, and the Skaw Karen to 24% in the Mon. All HbE genes were heterozygous (AE). Differences in HbE prevalence among the studied ethnic groups indirectly documents that ancestries and evolutionary forces, such as drift and admixture, are the important factors in the persistence of HbE distribution in northern Thailand. Furthermore, the presence of HbE in groups of northern Thailand had no effect on the *in vitro* infectivity and proliferation of *Plasmodium falciparum*, nor the production of hemozoin, a heme crystal produced by malaria parasites, when compared to normal red-blood-cell controls. Our data may contribute to a better understanding on the persistence of HbE among ethnic groups and its association with malaria.

## Introduction

The hemoglobin E (HbE) variant is caused by a genetic mutation at the codon 26 of the β-globin gene, changing an amino acid GAG (glutamine) in the normal hemoglobin A molecule (A) to AAG (lysine) [1]. HbE carriers usually have no clinical symptoms, but in combination with thalassemia (HbE/ β-thalassemia), may develop a mild to severe chronic anemia. The frequency of HbE varies from region to region and differs among different ethnic groups [2]. High prevalence of HbE is reported in various distinct groups in Southeast Asia, ranging from 5% to 10% in the region overall, and as high as 50% in some groups of Cambodia and Thailand [3].

Malaria, a parasitic disease caused by protozoa in the Genus *Plasmodium*, remains one of the most life-threatening disease in tropical countries [4]. Among five species that can infect humans, *P*. *falciparum* is the most lethal species causing malaria-related deaths. The association of various hemoglobinopathies with protection from malaria was systematically reviewed in a meta-analysis of 62 studies, revealing that most do protect against severe malaria symptoms [5], with variation in the degree of protection against mild or asymptomatic malaria infection. Paradoxically, the meta-analysis of two HbE studies suggested HbE does not confer protection to severe nor mild malaria but not in the other two independent studies [6,7]. Most of these studies focused on the major hemoglobinopathies, while HbE is rare and less is known about its influence on malaria.

Various parts of northern Thailand, including the malaria-endemic area along the Salween River, which forms the frontier between Myanmar and northwestern Thailand, and the lowland Chiang Mai-Lamphun basin, have been occupied for hundreds or even thousands of years by several ethnic groups [8], some of whom are often referred to as “hill tribes”, while some live in lowlands. Both are quite genetically distinct from the general Thai population [9,10]. Although the prevalence of HbE was approximately 12% in the general Thai population in 5 surveyed provinces of the 8 in northern Thailand [11], this proportion may not apply to these quite distinct ethnic groups because of sampling methods. To fill this gap in knowledge, we surveyed HbE prevalence among these ethnic groups of northern Thailand, and evaluated the association of the HbE gene with surrogate *in vitro* markers of protection against invasion of red blood cells (RBCs) by *P*. *falciparum*, such as the proportion of red blood cells infected *in vitro*, and the quantity of hemozoin, a product of hemoglobin digestion by the parasite. The data obtained may be helpful in understanding genetics protections against malaria.

## Materials and Methods

### Study populations

The study was approved by the Human Experimentation Committee, of the Research Institute for Health Sciences, Chiang Mai University, Thailand. Among a total of 18 recognized ethnic groups in northern Thailand [8,12], we sampled 10 villages composed of 8 of these groups (Shan, Padong Karen, Lawa, Pwo Karen, Skaw Karen, Khon Muang, Lue, and Mon). Five villages were selected for having the highest period prevalence of malaria infection in blood-smear surveys in 2007–2012 (ranging from 1.56% to 17.5%), and five for having the lowest prevalence (0 to 0.25%) [13]. Volunteers were healthy subjects, over 20 years old, being in majority ethnic group with no ancestors from another group for 3 generations and were enrolled with written informed consent. We tried to enroll 30 subjects per village. Data were collected by research staff using form-based oral interviews of subjects for self-reported unrelated lineage, migration history and ethnicity for the three prior generations of ancedents, history of malaria and hematological diseases. Five milliliters of peripheral blood were collected into EDTA-anticoagulated sterile tubes and delivered within one day to the research laboratory.

### Hematological testing

HbE, α- and β-thalassemia traits were identified using cation exchange high performance liquid chromatography, using methods described elsewhere [14,15]. The α-thalassemia trait was confirmed by multiplex gap polymerase chain reaction [16,17], and glucose-6-phosphate dehydrogenase (G6PD) deficiency by fluorescent spot test [18]. Complete blood cell count was performed by automatic hematology analyzer (Beckman Coulter, CA, USA).

### *P*. *falciparum* invasion assay

The *P*. *falciparum* laboratory 3D7 strain was maintained in continuous culture under standard conditions [19]. RBCs from participants with HbE, and not G6PD deficiency, α- nor β-thalassemia were studied for parasite invasion in comparison with RBCs from those of the same ethnic groups without hemoglobin variants. The RBCs were separated from whole blood using CF11 cellulose column [20], washed twice with RPMI 1640, resuspended in culture media and counted. Each sample was assessed in duplicates.

Mature schizonts were obtained by gradient centrifugation over 60% Percoll (GE Healthcare Life Science, Buckinghamshire, England) [19]. Isolated schizont-infected RBCs at 1x10^7^ cells was added into 10x10^8^ RBCs (1% parasitemia) and cultured for 96 hours. The percentages of infected RBCs on blood smears were determined by the average count of two human microscopists.

### Hemozoin assay

After 96 hours of incubation, formation of hemozoin was determined by methods described elsewhere [21,22]. Briefly, cultured RBCs were serially diluted to different dilutions, controlled by culture without parasite addition. After two cycles of centrifugation, discarding supernatant, adding 2.5% sodium dodecyl sulfate (SDS) in 0.1M NaHCO_3_, and incubating at room temperature for 20 minutes, 100 μl of 2.5% SDS in 1M NaHCO_3_ was added and then incubated for 30 minutes and absorbance measured at 405 nm and 750 nm on a Spectra MR plate reader (Dynex Technology, VA, USA).

### Statistical analysis

Correlations between each pair of parameters and *p*-values were calculated by using the R statistical programming language version 3.2 (The R Project for Statistical Computing (http://www.r-project.org). Analysis of variance (ANOVA) was tested by STATISTICA (StatSoft, OK, USA).

## Results

### Prevalence of variant genetic traits

Eight of the selected villages were in the Salween River area in northern and southern Mae Hong Son province, while two were in the Chiang Mai-Lamphun basin in both Chiang Mai and Lamphun provinces. Although the target sample size was 30 subjects per village, in Jong Kham (Shan), Huay Pu Kaeng (Padong Karen), Um Da Neur (Skaw Karen), Nong Du (Mon) and Luang Nuer (Lue) villages there were insufficient number of unrelated persons to satisfy this eligibility criterion. Thus, a total of 269 samples from 114 male and 155 female volunteers were obtained from the 10 villages.

**Fig 1:**
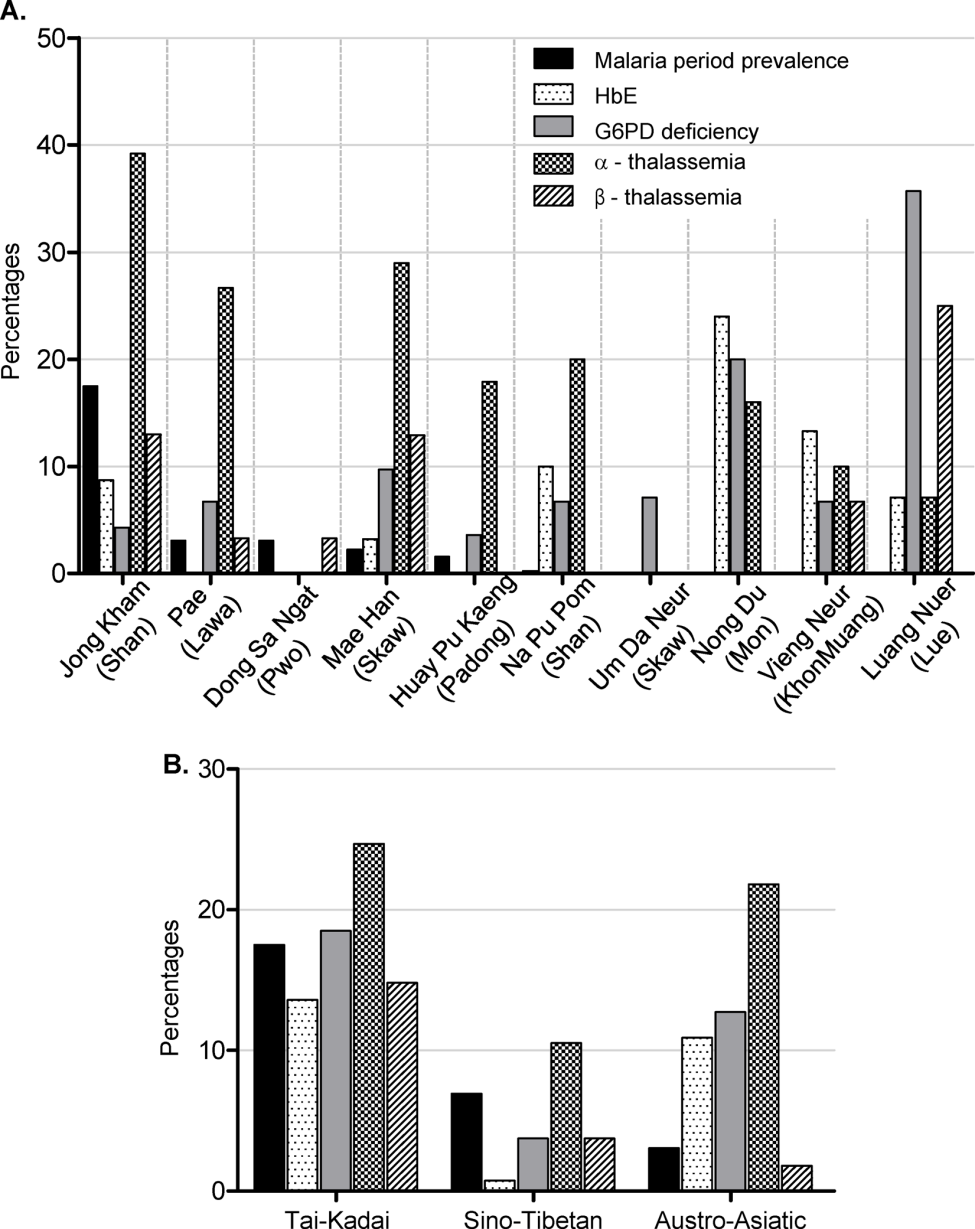
Proportions of studied characteristics (malaria prevalence, HbE, G6PD deficiency, α-thalassemia, β-thalassemia), by village and ethnic group (A) and by linguistic family (B).

HbE was detected in 6.7% (18/269) of the subjects (Table 1). The HbE genes of all 18 individuals were heterozygous (AE). The prevalence of HbE carriers ranged from 0 to 24% among the 10 villages, with the highest village Nong Du (Mon) (Fig 1A). Among the hemoglobin variants studied, α-thalassemia was most frequent (46/269, 17.1%), followed by G6PD deficiency (27/269, 10.0%), β-thalassemia (18/269, 6.7%), and HbE (18/269, 6.7%) (Table 1).

**Table 1:** Villages study, ranked by high and low period prevalence of malaria between 2007 through 2012 (selection criteria for study), from details on study participants, their localities, the percentages of malaria reported, ethnic group, linguistic families, number of sample in each groups, the number (and percentages) of hemoglobin disorder observed.

						No. of hemoglobin disorder observed (%)				
Village (District, Province)	Locality (Latitude °N/ Longitude °E)	Malaria prevalence	Ethnic group	Linguistic family	No. samples (M/F)	HbE	G6PD def	α-thal	β-thal	Total
**Jong Kham** (Muang, Mae Hong Son)	19°29′/97°96′	17.5	Shan	Tai-Kadai	23 (7/16)	2 (8.7)	1 (4.3)	9 (39.2)	3 (13)	**15 (65.2)**
**Pae** (Mae Sa Rieng, Mae Hong Son)	18°16′/97°94′	3.08	Lawa	Austro-Asiatic	30 (15/15)	0 (0)	2 (6.7)	8 (26.7)	1 (3.3)	**11 (36.7)**
**Dong Sa Ngat** (Mae Sa Rieng, Mae Hong Son)	18°15′/97°93′	3.08	Pwo Karen	Sino-Tibetan	30 (12/18)	0 (0)	0 (0)	0 (0)	1 (3.3)	**1 (3.3)**
**Mae Han** (Mae Sa Rieng, Mae Hong Son)	18°20′/97°88′	2.25	Skaw Karen	Sino-Tibetan	31 (16/15)	1 (3.2)	3 (9.7)	9 (29)	4 (12.9)	**17 (54.8)**
**Huay Pu Kaeng** (Muang, Mae Hong Son)	19°14′/97°93′	1.56	Padong Karen	Sino-Tibetan	28 (13/15)	0 (0)	1 (3.6)	5 (17.9)	0 (0)	**6 (21.4)**
**Na Pu Pom** (Pang Ma Pha, Mae Hong Son)	19°62′/98°11′	0.25	Shan	Tai-Kadai	30 (5/25)	3 (10)	2 (6.7)	6 (20)	0 (0)	**11 (36.7)**
**Um Da Neur** (Sob Mei, Mae Hong Son)	18°01′/97°98′	0.04	Skaw Karen	Sino-Tibetan	14 (9/5)	0 (0)	1 (7.1)	0 (0)	0 (0)	**1 (7.1)**
**Nong Du** (Pa Sang, Lamphun)	18°52′/98°89′	0	Mon	Austro-Asiatic	25 (13/12)	6 (24)	5 (20)	4 (16)	0 (0)	**15 (60.0)**
**Vieng Neur** (Pai, Mae Hong Son)	19°44′/98°50′	0	Khon Muang	Tai-Kadai	30 (13/17)	4 (13.3)	2 (6.7)	3 (10)	2 (6.7)	**11 (36.7)**
**Luang Nuer** (Doi Sa Ked, Chiang Mai)	18°89′/99°12′	0	Lue	Tai-Kadai	28 (11/17)	2 (7.1)	10 (35.7)	2 (7.1)	7 (25)	**21 (75.0)**
	**Total**				**269**	**18 (6.7)**	**27 (10.0)**	**46 (17.1)**	**18 (6.7)**	

When all four investigated traits were combined, it was found that the Luang Neur (Lue) had the highest (21/28, 75%), while the Dong Sa Ngat (Pwo Karen) had the lowest (1/30, 3.3%) prevalence of hemoglobinopathies. There was no statistical correlation between the HbE and malaria prevalence for each ethnic population. A slightly significant correlation (0.01 < *p* <0.05) was detected between α-thalassemia and malaria prevalence, and G6PD deficiency and β-thalassemia prevalence (Table 2).

**Table 2:** Calculated correlations (below the diagonal) and their probability *p* values (above the diagonal) between the percentages of malaria prevalence, HbE, G6PD deficiency, α-, and β-thalassemia. *p* < 0.05 was considered statistical significant (bold letter).

	Malaria prevalence	HbE	G6PD deficiency	α-thalassemia	β-thalassemia
**Malaria prevalence**		0.870	0.389	**0.037**	0.485
**HbE**	-0.059		0.252	0.755	0.984
**G6PD deficiency**	-0.306	0.399		0.646	**0.046**
**α-thalassemia**	0.662	0.113	-0.165		0.656
**β-thalassemia**	0.250	0.007	0.639	0.161	

When populations were grouped according to their linguistic families, high HbE prevalence was observed in the Tai-Kadai (11/111, 10%) and the Austro-Asiatic (6/55, 11%) linguistic families, while the Sino-Tibetan showed low HbE prevalence (1/103, 1%). The Sino-Tibetan population also showed low frequency of G6PD deficiency (5/103, 5%) and β-thalassemia (5/103, 5%). α-thalassemia was the most prevalent hemoglobin disorder observed for all linguistic families, ranged from 13.6% (14/103) in the Sino-Tibetan to 22% (12/55) in the Austro-Asiatic (Fig 1B).

### In vitro assay for *P*. *falciparum* invasion

**Fig 2:**
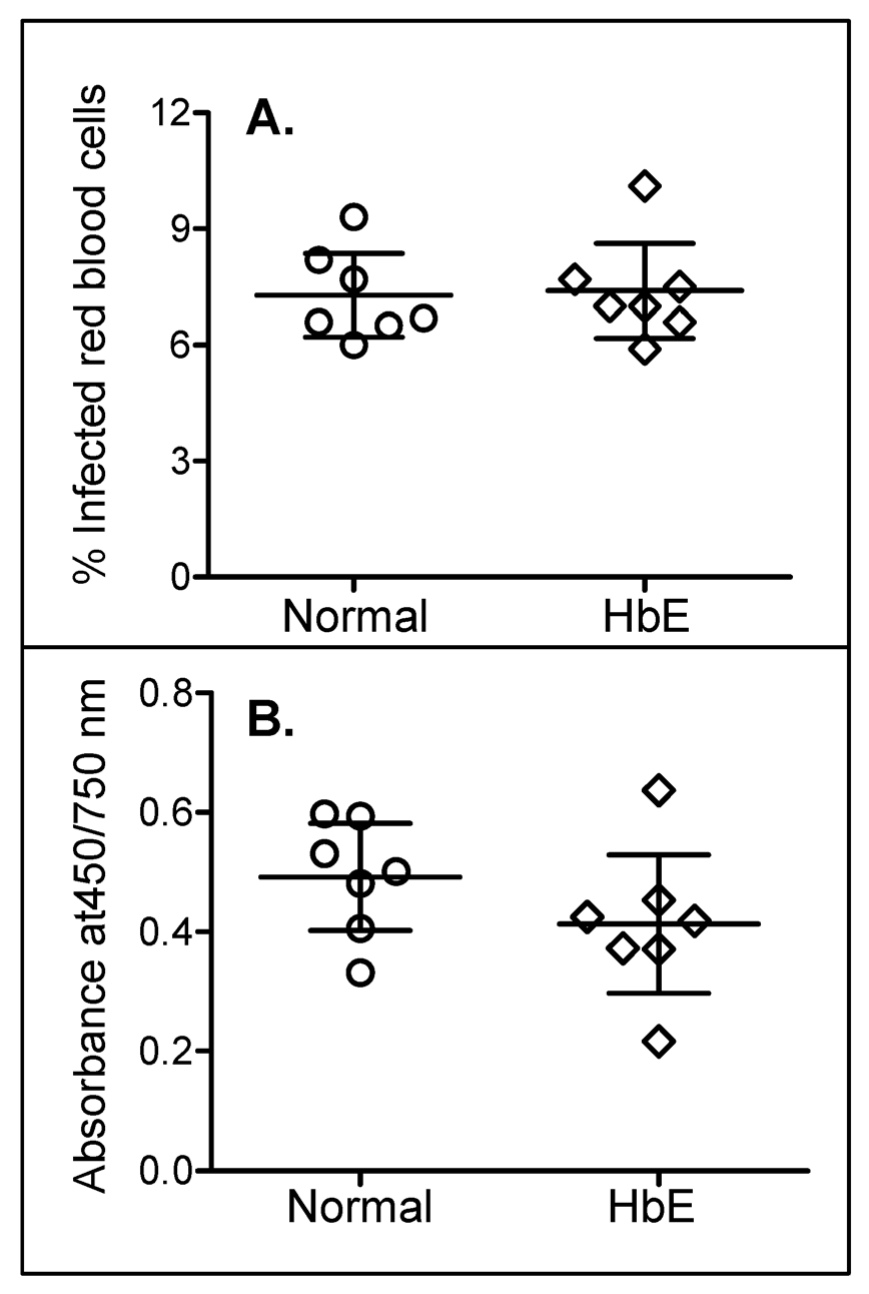
HbE did not have an inhibitory effect on *P*. *falciparum* invasion *in vitro* or the production of hemozoin. (A) The percentages of infected red blood cells and (B) the absorbance of hemozoin at 450/750 nm after 96 hours incubation of red blood cells from normal (circles) or HbE (diamonds) participants with *P*. *falciparum*. Each symbol represents each individual participant. Solid lines show the mean with standard deviations of each group.

After exclusion of G6PD deficiency and thalassemia, 7 HbE carriers (4 Khon Muang, 2 Mon, and 1 Lue) were willing to participate in the invasion assay study. After 96 hours of incubation RBCs and *P*. *falciparum*, the mean percentages (± SD) of infected RBCs in HbE carrier group (7.3 ± 1.12) was not statistically different from controls who were the same ethnic group with no hemoglobin disorder (7.4 ± 1.33) (Fig 2A). There was also no statistical difference of the absorbance of hemozoin when *P*. *falciparum* were cultures in HbE RBCs (O.D. 0.413 ± 1.33) or normal RBCs (O.D. 0.492 ± 0.097) (Fig 2B). When combined data from two groups, there was a positive correlation between % infected red blood cells and the absorbance of hemozoin (r = 0.53, *p* = 0.051, data not shown).

## Discussion

Our survey of HbE prevalence among ethnic groups residing in malaria-endemic area of northern Thailand found 6.7% of HbE carrier, which is slightly lower than a previous observation in the Northern Thai people [11]. The prevalence found in our study was in the average range (5–10%) reported for Southeast Asian populations [3]. However, there were differences in HbE prevalence among the studied ethnics, suggesting different genetic composition that may be a consequence from different common ancestors and their evolutionary process. Moreover, dissimilarities in HbE prevalence of the same ethnic groups from different villages indirectly documents that evolutionary forces, such as drift and admixture, may be important factors in the persistence of HbE prevalence within each ethnic inhabitant of northern Thailand.

The Nong Du (Mon) of Lamphun province exhibited the highest frequency of HbE among our study populations. The Mon was an indigenous ethnic group who established their civilization along the Ping River of northern Thailand during the 8^th^ to 13^th^ century, then fallen under the Tai’s invasion [23]. This was interesting in that the HbE prevalence was high in the Nong Du (Mon) but was not observed in the Pae (Lawa) who used to settle nearby at the same period of time. This observation suggests that the Nong Du (Mon) might carry HbE variants prior to migrating into northern Thailand.

During the prehistoric and proto-historic periods, the Mon ethnic group and their Austro-Asiatic speaking relatives occupied the broad areas where are now Cambodia, Northeast/Central Thailand, and Southern Myanmar [8]. Several studies have reported high prevalence of HbE in these populations [3] or in ethnic groups who had relationship with them [24]. Noteworthy, the Mon had dispersed to many regions of Thailand since the prehistoric time and many of them have intermarried with different ethnic groups. There may be the relationship between HbE frequency distribution and the Mon migration which need to be clarified by investigating the HbE prevalence in the Mon living in different regions of Thailand.

Although, many findings have pointed out the importance of HbE against malaria infection, there was no significant correlation between HbE prevalence and malaria incidence reported in ethnic groups of northern Thailand. However, this result should be interpreted with caution as there were a few limitations in our study. First, although most of our selected populations are settled in their present localities possibly since hundreds of years ago, members of the Sino-Tibetan linguistic family, i.e. the Dong Sa Ngat (Pwo Karen) and the Huay Pu Keang (Padong Karen), are still wandering around the border between Thailand and Myanmar (personal interview with villagers). Their recent subdivision populations or founder effect, which is a shift in allelic frequency due to the creation of new and isolated populations, may have more impact on the frequency of HbE than malaria endemicity. The relative lower HbE, G6PD deficiency, and thalassemia frequencies in the Sino-Tibetan than the Tai-Kadai or Austro-Asiatic was corresponding to this founder scenario. Secondly, owing to the fact that most of the ethnic people of northern Thailand tend to practice endogamous marriage, collection of unrelated samples was limited and small sample size from each village was analyzed. Thus, due to the recent demography and small sampling number, further investigations are needed to explore the relationship between HbE prevalence and malaria incidence of ethnic population in northern Thailand.

Taking into consideration of sufficient sample numbers by grouping study populations according to their linguistic origins, some speculations for the HbE and malaria relationship could be evaluated. The high HbE prevalence in the Austro-Asiatic family is in confronting with the malaria prevalence which is consistent with the Haldane’s hypothesis of heterozygous advantage selection against malaria [25]. Though the Tai-Kadai linguistic family also showed high frequency of HbE but it seems that this family was not in the selection by malaria since the percentages of HbE and malaria prevalence were about the same. However, the observation of HbE in the Tai-Kadai family might be ambiguous as one of the ethnic groups, the Vieng Nuer (Khon Muang), is genetically conglomerate population between the Mon and the Tai [26]. Relatively high HbE prevalence observed in this population might not be the result of HbE and malaria selection but might be due to HbE variants gene flow from their Mon parental population.

Analysis of various hemoglobinopathies indicates that hemoglobin mutations confer protection against severe malaria, but differ in the degree of protection against uncomplicated malaria and asymptomatic parasitemia [5]. Most observations on HbE and association with malaria were obtained from clinical studies evaluating severity of malaria and the presence of HbE [5,6,27,28,29,30,31]. We directly evaluated the growth of *P*. *falciparum in vitro* but no inhibitory effect was found. This was consistent with the observations in *P*. *falciparum* patients which reported no association between HbE and malaria [28,29]. However, our data were in contrast with an *in vitro* study in different Thai populations which reported inhibitory effect of HbE *in vitro* [7]. The distinction of observations could be due to different study populations as well as different *P*. *falciparum* strains used for invasion assay. In addition, the ability to proliferate in both normal and hemoglobinopathic RBCs of different strains of *P*. *falciparum* can differ substantially [32]. However, we did not assess the impact of HbE on the invasion of *P*. *falciparum* field isolates, therefore bias due to strain specificity could not be ruled out. It is also worth to take note that due to limitation of light microscope which provides 2-dimentional images of infected red blood cells, we did not enumerate the number of merozoites released from an individual schizont as it could underestimate merozoite number. Further investigation on larger ethnic populations, invasion of *P*. *falciparum* field isolates, and enumeration of merozoite number will be of great interest and need to be addressed in the future.

Hemozoin or malaria pigment is a product from the detoxification process used by malaria parasites to convert toxic free heme, which is an outcome of hemoglobin digestion, into an insoluble crystalline form [33]. It is involved in pathophysiology of the disease and induction of the host immune responses. Understanding of abnormal hemoglobin and the formation of hemozoin is still limited. Although we did not find differences in hemozoin formation between HbE and normal RBCs, it was not conclusive from our study that abnormal hemoglobin does not have an effect on hemozoin formation due to small sample numbers. A previous study demonstrated that substitution of heme with heme analogs resulted in the inhibition of hemozoin formation, but did not inhibit parasite growth[34]. The effect of abnormal hemoglobin on hemozoin needs to be explored with respect to the development of malaria treatment.

In conclusion, we reported the overall HbE prevalence of 6.7% among ethnic groups residing in northern Thailand, varying among groups. Ancestry, genetic drift, and admixture have impact on the HbE distribution. Despite of small sample numbers, our data suggest that HbE does not have an effect on *P*. *falciparum* growth and hemozoin formation.
