# Correction: Hemoglobin E Prevalence among Ethnic Groups Residing in Malaria-Endemic Areas of Northern Thailand and Its Lack of Association with *Plasmodium falciparum* Invasion *In Vitro*

**DOI:** 10.1371/journal.pone.0163430

**Published:** 2016-09-15

**Authors:** Pathrapol Lithanatudom, Jiraprapa Wipasa, Pitsinee Inti, Kriangkrai Chawansuntati, Saovaros Svasti, Suthat Fucharoen, Daoroong Kangwanpong, Jatupol Kampuansai

The values for columns “No. samples (M/F),” and “No. of hemoglobin disorder observed (%)” for rows “Pae” and “Dong Sa Ngat” are incorrectly switched. Please see the corrected Table 1 below.

**Table 1:** Villages study, ranked by high and low period prevalence of malaria between 2007 through 2012 (selection criteria for study), from details on study participants, their localities, the percentages of malaria reported, ethnic group, linguistic families, number of sample in each groups, the number (and percentages) of hemoglobin disorder observed.

						No. of hemoglobin disorder observed (%)				
Village (District, Province)	Locality (Latitude °N/ Longitude °E)	Malaria prevalence	Ethnic group	Linguistic family	No. samples (M/F)	HbE	G6PD def	α-thal	β-thal	Total
**Jong Kham** (Muang, Mae Hong Son)	19°29′/97°96′	17.5	Shan	Tai-Kadai	23 (7/16)	2 (8.7)	1 (4.3)	9 (39.2)	3 (13)	**15 (65.2)**
**Pae** (Mae Sa Rieng, Mae Hong Son)	18°16′/97°94′	3.08	Lawa	Austro-Asiatic	30 (12/18)	0 (0)	0 (0)	0 (0)	1 (3.3)	**1 (3.3)**
**Dong Sa Ngat** (Mae Sa Rieng, Mae Hong Son)	18°15′/97°93′	3.08	Pwo Karen	Sino-Tibetan	30 (15/15)	0 (0)	2 (6.7)	8 (26.7)	1 (3.3)	**11 (36.7)**
**Mae Han** (Mae Sa Rieng, Mae Hong Son)	18°20′/97°88′	2.25	Skaw Karen	Sino-Tibetan	31 (16/15)	1 (3.2)	3 (9.7)	9 (29)	4 (12.9)	**17 (54.8)**
**Huay Pu Kaeng** (Muang, Mae Hong Son)	19°14′/97°93′	1.56	Padong Karen	Sino-Tibetan	28 (13/15)	0 (0)	1 (3.6)	5 (17.9)	0 (0)	**6 (21.4)**
**Na Pu Pom** (Pang Ma Pha, Mae Hong Son)	19°62′/98°11′	0.25	Shan	Tai-Kadai	30 (5/25)	3 (10)	2 (6.7)	6 (20)	0 (0)	**11 (36.7)**
**Um Da Neur** (Sob Mei, Mae Hong Son)	18°01′/97°98′	0.04	Skaw Karen	Sino-Tibetan	14 (9/5)	0 (0)	1 (7.1)	0 (0)	0 (0)	**1 (7.1)**
**Nong Du** (Pa Sang, Lamphun)	18°52′/98°89′	0	Mon	Austro-Asiatic	25 (13/12)	6 (24)	5 (20)	4 (16)	0 (0)	**15 (60.0)**
**Vieng Neur** (Pai, Mae Hong Son)	19°44′/98°50′	0	Khon Muang	Tai-Kadai	30 (13/17)	4 (13.3)	2 (6.7)	3 (10)	2 (6.7)	**11 (36.7)**
**Luang Nuer** (Doi Sa Ked, Chiang Mai)	18°89′/99°12′	0	Lue	Tai-Kadai	28 (11/17)	2 (7.1)	10(35.7)	2 (7.1)	7 (25)	**21 (75.0)**
	**Total**				**269**	**18 (6.7)**	**27 (10.0)**	**46 (17.1)**	**18 (6.7)**	

There are errors in Fig 1 for “Pae (Lawa)” and “Dong Sa Ngat (Skaw).” Please see the correct Fig 1 here.

**Fig 1:**
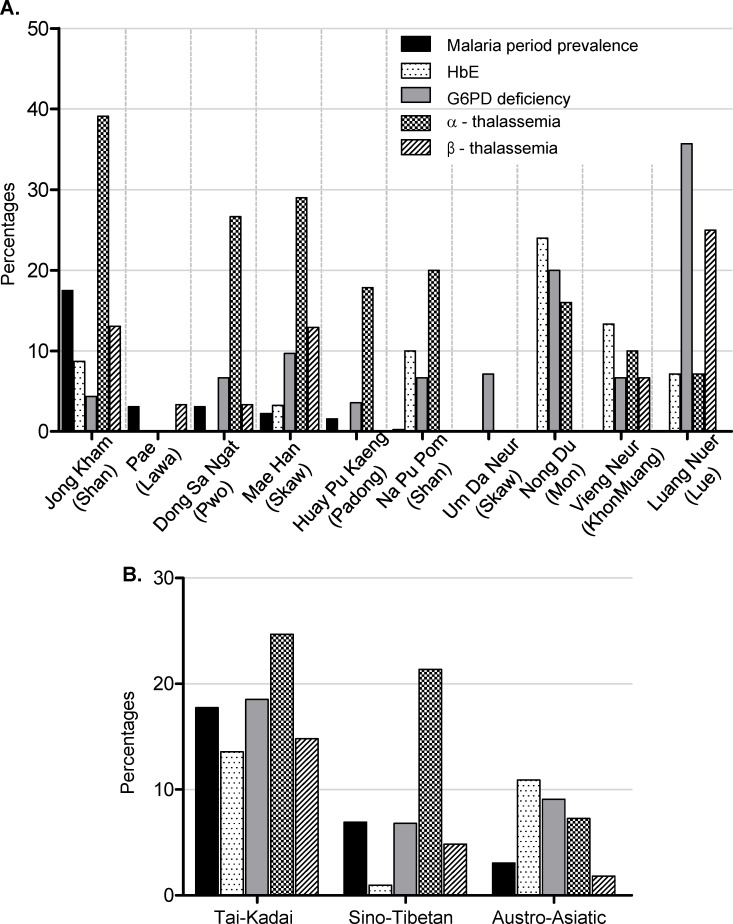
**Proportions of studied characteristics (malaria prevalence, HbE, G6PD deficiency, α-thalassemia, β-thalassemia), by village and ethnic group (A) and by linguistic family (B)**.

There is an error in the first sentence of the third paragraph in the Results section. The correct sentence is: When all four investigated traits were combined, it was found that the LuangNeur (Lue) had the highest (21/28, 75%), while the Pae (Lawa) had the lowest (1/30, 3.3%) prevalence of hemoglobinnopathies.

There is an error in the second sentence of the fourth paragraph in the Results section. The correct sentence is: The Sino-Tibetan population also showed low frequency of G6PD deficiency (7/103, 7%) and **β**–thalassemia (5/103, 5%).

There are errors in the third sentence of the fourth paragraph in the Results section. The correct sentence is: **α**-thalassemia was the most prevalent haemoglobin disorder observed, ranged from 7.2% (4/55) in the Austro-Asiatic to 24.7% (20/111) in the Tai-Kadai (Fig 1B).

There is an error in the the fifth sentence in the fourth paragraph in the Discussion section. The correct sentence is: The relative lower HbE frequencies in the Sino-Tibetan than the Tai-Kadai or Austro-Asiatic was corresponding to this founder scenario.
